# The role of the GABAA receptor Alpha 1 subunit in the ventral hippocampus in stress resilience

**DOI:** 10.1038/s41598-019-49824-4

**Published:** 2019-09-18

**Authors:** Z. Ardi, A. Richter-Levin, L. Xu, X. Cao, H. Volkmer, O. Stork, G. Richter-Levin

**Affiliations:** 1 Sagol Department of Neuroscience, University of Haifa, Haifa, 3498838 Israel; 2 Present Address: Department of Behavioral Sciences, Kinneret Academic College, Sea of Galilee, Tiberias, Israel; 3 Sagol Center for Brain and Mind, Baruch Ivcher School of Psychology, Interdisciplinary Center (IDC), Herzliya, Israel; 4 The Integrated Brain and Behavior Research Center (IBBR), University of Haifa, Haifa, 3498838 Israel; 5 Key Laboratory of Animal Models and Human Disease Mechanisms, and Laboratory of Learning and Memory, Kunming Institute of Zoology, The Chinese Academy of Sciences, Kunming, 650223 China; 6 CAS Centre for Excellence in Brain Science and Intelligent Technology, Shanghai, 200031 China; 7 University of Chinese Academy of Sciences, Beijing, 100049 China; 8 Key Laboratory of Brain Functional Genomics, MOE&STCSM, East China Normal University, Shanghai, 200062 China; 9 Deptartment Molecular Biology, Natural and Medical Sciences Institute at the University of Tübingen, Markwiesenstr. 55, 72770 Reutlingen, Germany; 10 Department of Genetics & Molecular Neurobiology, Institute of Biology, Otto-von-Guericke University Magdeburg, Magdeburg, 39120 Germany; 11 Department of Psychology, University of Haifa, Haifa, 3498838 Israel

**Keywords:** Post-traumatic stress disorder, Stress and resilience, Post-traumatic stress disorder

## Abstract

Pre-pubertal stress increases post-traumatic stress disorder (PTSD) susceptibility. We have previously demonstrated that enriched environment (EE) intervention immediately after pre-pubertal stress protects from the effects of trauma in adulthood. Here, we examined whether exposure to EE would also be beneficial if applied after exposure to trauma in adulthood. We have recently shown that exposure to juvenile stress and under-water trauma (UWT) is associated with increased expression of GABA_A_ receptor subunit α1 in the ventral hippocampus. However, differentiating between affected and unaffected individuals, this increased expression was confined to stress-exposed, behaviorally unaffected individuals, suggesting upregulation of α1 expression as a potential mechanism of resilience. We now examined whether EE-induced resilience renders increased expression of α1 in the ventral hippocampus redundant when facing a trauma later in life. Adult rats were exposed to UWT, with pre-exposure to juvenile stress, and tested in the open field and elevated plus maze paradigms four weeks later. EE exposure during juvenility prevented pre-pubertal stress-induced vulnerability, but not if performed following UWT in adulthood. Furthermore, juvenile EE exposure prevented the trauma-associated increase in α1 expression levels. Our findings emphasize the importance of early interventions in order to reduce the likelihood of developing psychopathologies in adulthood.

## Introduction

Exposure to childhood stress is a well described risk factor of PTSD in adulthood^1–3^. Indeed, early adolescence (juvenility) constitutes a stress-sensitive period. Across species, including rats and humans, the juvenile brain is noticeably different from that of newborns, weanlings, or adults^4,5^. Accordingly, in rodents, we and others have demonstrated that juvenile (pre-pubertal) stress increases the risk for developing PTSD-related symptoms in adulthood^6–10^. While attention is directed towards possible mechanisms of induced vulnerability, it is important to consider the possibility that some brain responses to stress exposure may be adaptive, aiming to reduce the risk for PTSD later in life. In line with that, we have recently demonstrated that pharmacological treatment with fluoxetine during juvenility reduced the risk of developing symptoms in an animal model of PTSD^11^. By contrast, the treatment was not effective when applied in adulthood^9^. We further demonstrated that juvenile animals exposed to an enriched environment (EE) starting immediately after the exposure to pre-pubertal stress and lasting into adulthood were protected from the deleterious effects of a trauma in adulthood^12,13^. To examine whether EE could be as effective in treating PTSD as it is in preventing its development, we now examined whether a similar exposure to EE could also be effective if given in adulthood, from immediately after the exposure to the adulthood trauma.

Many studies suggest that alterations in GABA functioning are associated with stress and trauma^14–17^. Recently, we have shown that exposing animals to under-water trauma (UWT) with pre-exposure to juvenile stress was associated with increased expression of GABA_A_ receptor subunit α1, in the ventral but not dorsal hippocampus of exposed animals^18^. However, classification of the exposed population to affected and unaffected individuals by using behavioral profiling analysis revealed that the increased GABA_A_ receptor α1 expression was evident only in exposed, unaffected animals, indicating a resilience-associated expression regulation^18^.

In this study, we therefore first examined whether the exposure to juvenile stress combined with UWT would be associated with a similar increased expression of α1 in the ventral hippocampus. We further examined whether such an increased expression of α1 would indeed be restricted only to exposed, unaffected individuals. Finally, we analyzed the effects of exposure to EE, either during juvenility or only in adulthood, on both behavior and α1 expression in the ventral hippocampus.

## Material and Methods

### Animals

Male Sprague-Dawley rats were purchased (Envigo Laboratories, Jerusalem, Israel) at postnatal day (PND) 22, weighing 30–50 g. Animals were housed in groups of 4–5 rats per cage, with ad libitum access to food and water (22 ± 2 °C; 12:12 hours light-dark cycle). All experiments were carried out in accordance with the NIH guide for care and use of laboratory animals. All experiments were approved by the ethics committee of the University of Haifa. All experimental procedures and assessments were preformed in designated rooms away from the vivarium between 9 AM and 3 PM.

### Stress protocols

#### Juvenile stress (J) exposure

The current study followed the juvenile stress protocol as described before by Horovitz and colleagues^8^. Rats were exposed to three different stressors for three consecutive days (PND 27–29): Day 1, 10 minutes of forced swim stress; Day 2, elevated platform for 3 × 30 minutes (1-hour ITI in the home cage); Day 3, 2 hours in a restraint apparatus.

#### Odor and underwater trauma (UWT association learning)

The protocol was performed as previously described by Ardi and colleagues^18^. In order to associate the odor with the UWT, all rats were first habituated to the “association cage” (a standard plastic cage covered with a plastic lid) for three days (2 min per day). On the 4th day, following the 2 min habituation, all rats were exposed to vanilla odor (100 µl concentrated vanilla extract in 15 ml of distilled water) for 30 s inside the cage. Immediately after odor exposure, all rats except controls were exposed to the UWT, by placing them in a water-filled plastic tank, and after 5 s of free swimming, restraining them under water for 45 s using a metal net. Control rats, on the 4th day, were exposed to the odor without exposure to UWT.

### Enriched environment housing

Animals were housed together (4–5 rats per cage) in a large cage (80 × 80 × 50 cm^3^) and were provided with three differently shaped plastic containers, two tunnels, one colored platform, and two running wheels for four weeks. The objects and sawdust were changed twice a week.

### Behavioral assessments

Behavioral assessments in the open field and elevated plus maze paradigms took place four weeks after UWT and odor (or only odor) exposure and were conducted as described before^18^.

#### Odor re-exposure

In order to change the original context of the odor reminder, the habituation cage that was used for the odor-UWT association learning was filled with a different type of sawdust than the type used during learning and the walls of the habituation cage were covered with geometric shapes. In the behavioral assessment room, rats were first habituated for 2 min and then were exposed for 30 s to the same vanilla odor that was used for association learning (equal odor concentration). Immediately following odor exposure, rats were subjected to behavioral tests.

#### Open field (OF) test

Rats were placed in the corner of the OF (under dim red-light illumination), and were left to explore the arena for 5 min^6,18^.

*Elevated plus maze test (EPM)*^6,18^ was carried out 24 hours after the OF test. Rats were placed in the center of the maze (two opposing open arms/closed arms, under full light illumination), facing one of the open arms, and were left to explore the maze for 5 min.

Rat behavior in the OF and EPM was recorded and then analyzed using EthoVision XT8 video tracking system (Noldus, Wageningen, Netherlands).

### Experimental groups and design

**Figure 1:**
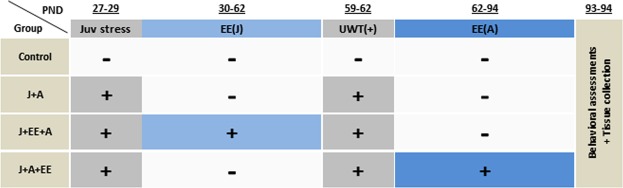
Experimental design. J + A rats were exposed to juvenile stress at PND 27–29 and to underwater trauma (UWT) and odor association learning at PND 59–62. J + EE + A and J + A + EE rats were exposed to the same stressors but were housed in environmentally enriched cages either in juvenility or in adulthood. Control rats were not exposed to juvenile stress, UWT, or environmental enrichment conditions.

The experimental design is depicted in Fig. 1. Following delivery and five days of acclimation period, rats were randomly assigned to the different experimental groups: (1) Control, n = 19; (2) Juvenile stress + UWT in adulthood (A) exposures (J + A, n = 16); (3) Juvenile stress + UWT exposures + enriched environment housing in juvenility (J + EE + A, n = 16); (4) Juvenile stress + UWT exposures + enriched environment housing in adulthood (J + A + EE, n = 17). All rats except controls were exposed to juvenile stress (PND 27–29), and on the following day, J + EE + A rats were transferred from standard housing conditions to EE housing conditions (PND 30–62), while all other groups were subjected to normal housing conditions. In adulthood (PND 59–62), all rats except controls were exposed to the UWT protocol. Control rats were exposed to the same behavioral procedure without the UWT exposure. Immediately following the day of UWT, J + EE + A rats returned to standard housing conditions and J + A + EE rats were subjected to EE conditions (PND 62–94). Four weeks after UWT (PND 93–94), rats were subjected to behavioral assessments in the open field and elevated plus maze. Immediately following the last behavioral assessment, rats were decapitated, and their brains were collected.

### Behavioral profiling

**Figure 2:**
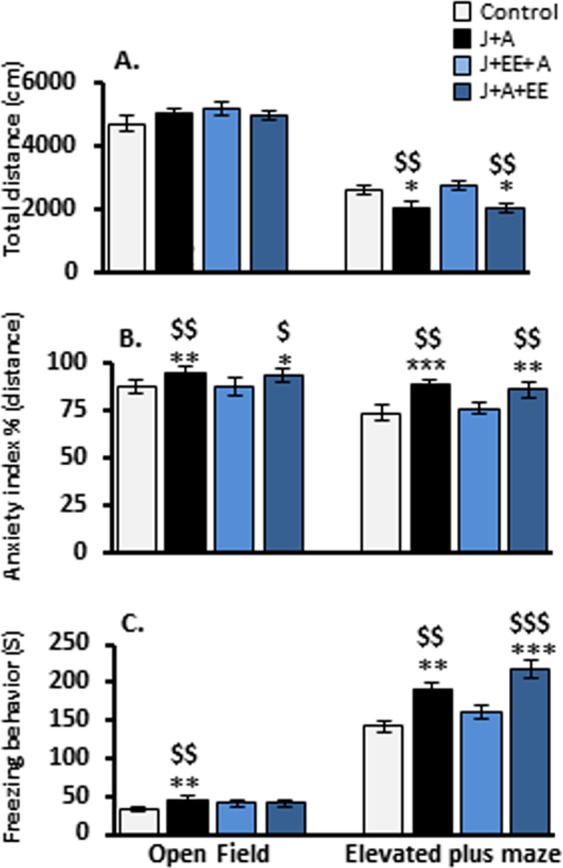
Exposure to enriched environment ameliorates the effects of stress if provided in juvenility, but not in adulthood. Group effects in the open field (OF) and elevated plus maze (EPM) paradigms. **(A**) Total distance. In the EPM, both J + A and J + A + EE rats were less active compared to control and J + EE + A rats. **(B**) Anxiety index. In the OF and in the EPM, both J + A and J + A + EE rats exhibited higher anxiety index compared to control and J + EE + A rats. **(C**) Freezing behavior. In the OF, J + A rats spent more time freezing compared to control and J + EE + A rats. In the EPM, both J + A and J + A + EE rats spent more time freezing compared to control and J + EE + A rats. All values are mean ± SEM. Significant difference compared to control: *p < 0.05, **p < 0.01, ***p < 0.001. Significant difference from J + EE + A: ^$^p < 0.05, ^$$^p < 0.01, ^$$$^p < 0.001.

In the current study, we adopted the behavioral profiling approach as previously described by Ardi and colleagues^18^. In order to build individual behavioral profiles based on performance in the OF and EPM tests, we first explored the distribution of different behavioral values in the control group while referring to the performance of the control group as representative of the normal population. We used standard deviations in order to determine cut-off values for each behavioral parameter examined (Fig. 2A). Later, we compared the performance of each animal in the experiment to the relevant distribution curve of the control group. Rats that exhibited values that were outside the range between the lower and upper cut-off values in at least four out of the six behavioral measurements were classified as “affected” (Fig. 2B).

### Western blot

Brain harvesting, tissue collection, and western blot analysis were performed as previously described^18^. Immediately after the last behavioral assessment (PND 94), brains were collected and snap frozen for biochemical analysis^16^. Briefly, frozen tissue punches (diameter: 1 mm; depth 1.5 mm) were collected bilaterally from the dorsal dentate gyrus (dDG) and the dorsal Cornu Ammonis 1 (dCA1; both starting −2.8 mm from Bregma, coronal) as well as the ventral DG and CA1 (vDG and vCA1; starting −7.6 mm from Bregma, horizontal orientation) according to the Paxinos and Watson brain atlas^19^.

Tissue samples were homogenized in 300 µl urea lysis buffer [1 mM EDTA, 0.5% Triton X, 6 M Urea, 100 μM PMSF; Sigma-Aldrich, St. Louis, MO] containing protease and phosphatase inhibitors (Complete Ultra and PhosStop tablets [Roche Diagnostics, Mannheim, Germany]). Samples containing 10 µg protein were loaded on a 12% SDS-polyacrylamide gel for electrophoresis, followed by semi-dry transfer to nitrocellulose membranes and blocking. Membranes were first incubated with primary antibodies (rabbit α GABA_A_ α1 1:2,500, Synaptic Systems, Göttingen, Germany; rabbit α GAPDH 1:2,000, Cell Signaling, Beverly, MA, USA; overnight at 4 °C), then with a complementary secondary antibody (α rabbit, polyclonal, 1:15,000). ECL Plus substrate (Advansta, Menlo Park, CA, USA) enabled chemiluminescence detection, and density of signals was analyzed with the Quantity One 1-D Analysis software. Ratios between optical density of target protein and control protein GAPDH were calculated for each sample and normalized first to a reference brain sample that was loaded on each gel for standardization across gels, and then to the mean density of the control group for each target and area.

### Statistical analysis

First, differences in behavior and protein expression levels between the different experimental groups were tested using two-way ANOVA, followed by Fisher’s protected least significant difference (PLSD) post hoc test. Second, after using the behavioral profiling, we used Pearson’s chi-squared test in order to calculate the distribution of affected vs. unaffected populations. Last, relying on the behavioral profiling classification to “unaffected” and “affected” sub-populations in each experimental group, we then re-analyzed the protein expression data using Multivariate analysis for testing the effects of group, behavioral profile, and their interaction for each brain target and region. Significant interactions were further analyzed using planned comparisons within the different experimental groups and profiles. Specifically, we used one-way ANOVA followed by Fisher’s PLSD post hoc test in order to test for differences between experimental groups in each behavioral profile (unaffected/affected animals), and two tailed t-tests in order to test for differences between affected and unaffected rats within each experimental group. In order to avoid type 1 errors, we used the sequential Bonferroni correction to correct for multiple comparisons^20^.

## Results

### Averaged group effects in the open field and the elevated plus maze following exposure to stress and enriched environment

In order to evaluate the anxiolytic effects of exposure to juvenile and adult stress and exposure to an enriched environment (EE) during juvenility or in adulthood, we first compared the average group effects in the open field (OF) and elevated plus maze (EPM) paradigms, measuring total distance, anxiety index, and freezing behavior. No effects were found in the OF in the total distance travelled (two-way ANOVA; J + A: F(3,67) = 1.22, n.s.; EE: F(3,67) = 0.288, n.s.). In the EPM, two-way ANOVA revealed a significant main effect for both J + A and EE exposure and the interaction between the two factors (F(3,67) = 4.94, p = 0.03; F(3,67) = 5.24, p = 0.008; F(3,67) = 4.41, p = 0.007, respectively). Further post hoc comparisons revealed a reduction in total activity in the EPM for both J + A and J + A + EE groups compared to control (p = 0.03, p = 0.021 respectively) and to J + EE + A rats (p = 0.008, p = 0.005, respectively; Fig. 2A).

Calculating the anxiety index (distance in safe zone/[distance in safe zone + distance in higher risk zone]), two-way ANOVA revealed significant main effects both in the OF (J + A: F(3,67) = 8.71, p = 0.004; EE: F(3,67) = 4.84, p = 0.011; Interaction: F(3,67) = 5.027, p = 0.003) and in the EPM (J + A: F(3,67) = 15.94, p = 0.000; EE: F(3,67) = 5.95, p = 0.004; Interaction: F(3,67) = 7.771, p = 0.000). Further post hoc comparisons revealed that in the OF both J + A and J + A + EE rats showed elevated anxiety levels compared to control (p = 0.004, p = 0.011, respectively) and J + EE + A rats (p = 0.006, p = 0.014, respectively). In the EPM, post hoc comparisons revealed that both J + A and J + A + EE rats showed elevated anxiety levels compared to control (p = 0.000, p = 0.002, respectively) and to J + EE + A rats (p = 0.002, p = 0.01, respectively; Fig. 2B).

Measuring freezing behavior as expressed by total freezing time, two-way ANOVA revealed significant main effects both in the OF (J + A: F(3,67) = 7.795, p = 0.007; EE: F(3,67) = 4.281, p = 0.018; Interaction: F(3,67) = 3.932, p = 0.012) and the EPM (J + A: F(3,67) = 10.942, p = 0.002; EE: F(3,67) = 10.224, p = 0.000; Interaction: F(3,67) = 11.652, p = 0.000). Further post hoc comparisons revealed that, in the OF, J + A rats showed increased freezing levels compared to control and J + EE + A rats (p = 0.007, p = 0.005, respectively). In the EPM, increased freezing levels were found for both J + A and J + A + EE groups compared to control (p = 0.002, p = 0.000, respectively) and J + EE + A rats (p = 0.009, p = 0.000, respectively; Fig. 2C).

### Assessing the prevalence of affected animals in the different groups by using the behavioral profiling approach

**Figure 3:**
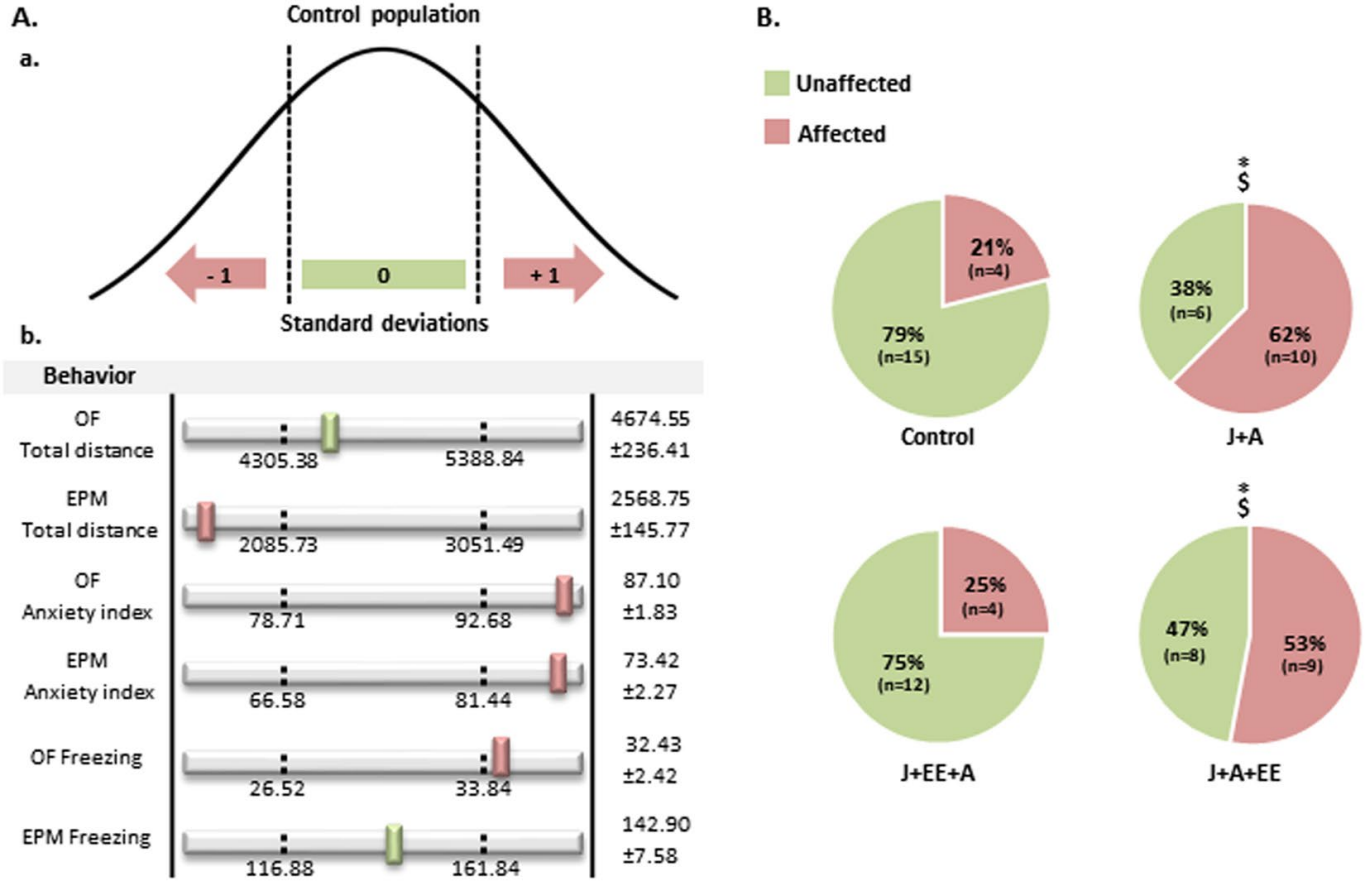
Assessing the prevalence of affected animals in the different groups by using the behavioral profiling approach. (**A**) Schematic representation of the behavioral profiling approach. (a) Representative distribution curve of a given behavior in the control group. Standard deviations were used for calculating cut-off values based on the relevant distribution curve of the control group. (b) Representative example of a behavioral profile of an affected animal. In order to be classified as affected, an animal must exhibit values outside the range of the cut-off values in at least four out of the six behavioral parameters. **(B**) Prevalence of affected animals in the different groups By using the behavioral profiling approach, a significant higher proportion of affected animals was detected among both J + A and J + A + EE rats compared to the proportion of affected animals among control and J + EE + A rats. All values are mean ± SEM. Significant difference from control: *p < 0.05. Significant difference from J + EE + A: ^$^p < 0.05.

Behavioral profiling analysis, which takes into consideration individual differences in responses to stress and trauma, enables the classification of affected and unaffected individuals in a trauma-exposed population^18,21^ (Fig. 3A). Following the characterization of individual animals as “affected” or “unaffected”, we re-analyzed the behavioral data presented in Fig. 2.

To determine the distribution of affected animals within the different groups, the number of individuals that exhibited values below the lower cut-off value or above the upper cut-off value in at least four out of the six behavioral parameters was compared between the different stress-exposure groups. Pearson χ^2^ analysis revealed a significant difference in the distribution of affected and unaffected individuals (χ^2^ likelihood ratio(3) = 9.13, p = 0.021; Fig. 3B).

Specific comparisons between groups using Pearson χ^2^ analysis revealed an increased prevalence of affected rats in the J + A group compared to control and J + EE + A groups (χ^2^ likelihood ratio(1) = 6.2, p = 0.013; χ^2^ likelihood ratio(1) = 4.69, p = 0.033, respectively). A similar pattern of different distribution rates of affected animals was also found in J + A + EE rats compared to control and J + EE + A populations (χ^2^ likelihood ratio(1) = 4.02, p = 0.047; χ^2^ likelihood ratio(1) = 3.94, p = 0.05 respectively). There was no significant difference between the J + A and J + A + EE groups.

### Expression levels of GABA_A_ receptor α1 subunit in dorsal and ventral hippocampal subregions following stress exposure and enriched environment intervention

**Figure 4:**
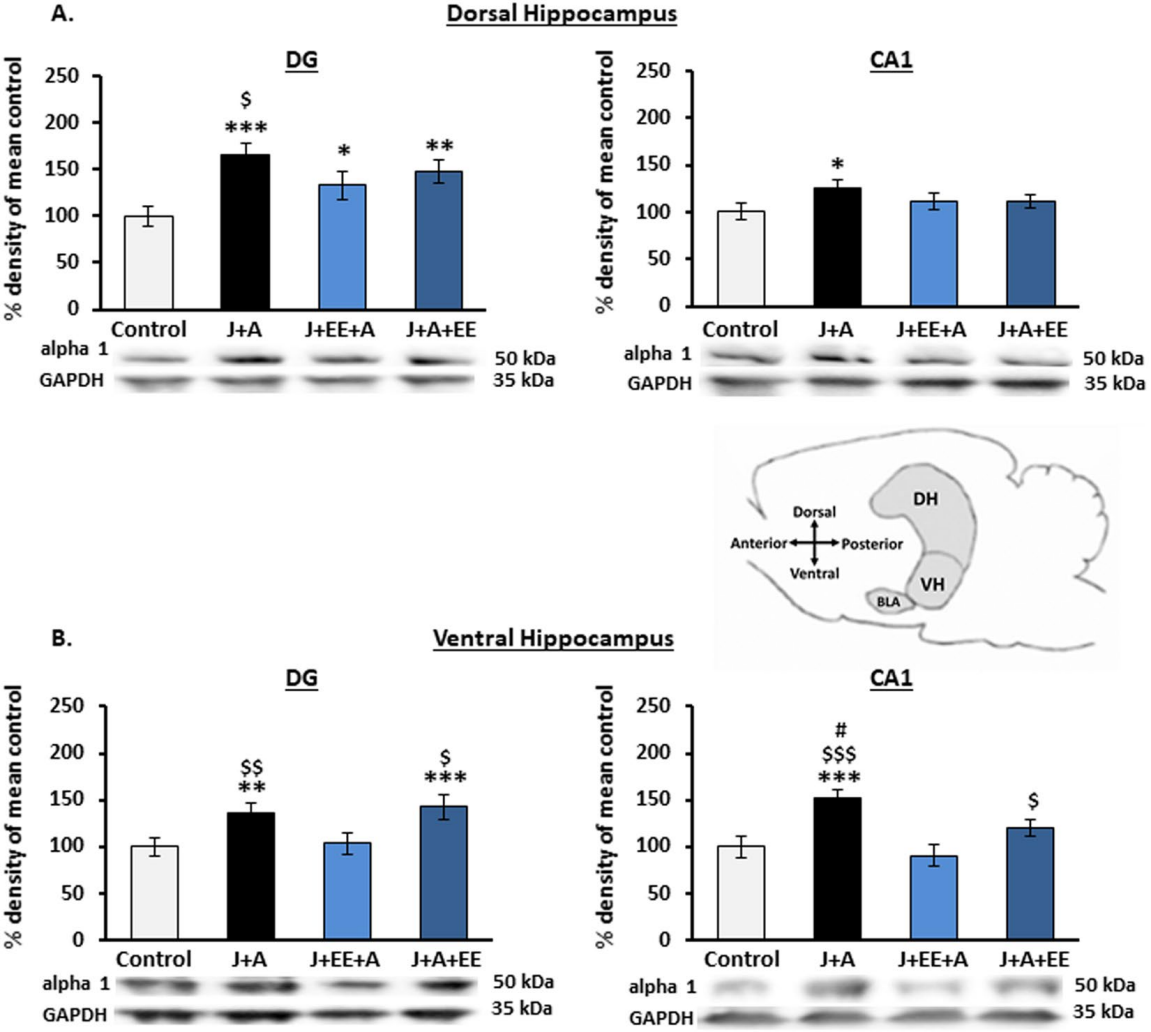
Alterations in expression levels of GABA_A_ receptor α1 subunit in dorsal and ventral hippocampal subregions following stress exposure and environmental enrichment. **(A**) Dorsal hippocampus. In the dDG, expression levels of GABA_A_ α1 were increased in all groups compared to control rats. In the dCA1, α1 expression levels were increased only in J + A rats compared to control. **(B**) Ventral hippocampus. In the vDG, both J + A and J + A + EE rats showed higher expression levels of GABA_A_ receptor α1 compared to control and J + EE + A rats. In the vCA1, while J + A rats showed higher α1 expression levels compared to all other groups, J + A + EE rats showed increased α1 expression compared to J + EE + A rats. All values are % density of mean control (mean ± SEM). Significant difference from control: *p < 0.05, **p < 0.01, ***p < 0.001. Significant difference from J + EE + A: ^$^p < 0.05, ^$$^p < 0.01, ^$$$^p < 0.001. Significant difference from J + A + EE: # p < 0.05. Sample blots are line blots from the same gel.

Following previous findings, indicating increased expression levels of the GABA_A_ receptor α1 subunit in the ventral hippocampus of stress-exposed animals^16^, we examined the effects of EE on the expression levels of GABA_A_ α1 in the dorsal and ventral hippocampus in the groups exposed to stress. In the dorsal hippocampus (Fig. 4A), two-way ANOVA revealed a significant effect for both J + A and EE exposure and their interaction on GABA_A_ α1 expression levels in the dDG (J + A: F(3,67) = 21.24, p = 0.000; EE: F(3,67) = 3.57, p = 0.034; Interaction: F(3,67) = 7.556, p = 0.000). In the dCA1, two-way ANOVA revealed a significant effect for the J + A exposure (F(3,67) = 5.193, p = 0.026) and a borderline significant effect for both EE exposure and the J + A and EE interaction (EE: F(3,67) = 2.56, p = 0.081; Interaction: F(3,67) = 2.462, p = 0.071). Further post hoc comparisons revealed that, in the dDG, both J + A, J + EE + A, and J + A + EE showed increased α1 expression levels as compared to control (p = 0.000, P = 0.038, p = 0.003, respectively). In addition, J + A rats showed increased α1 expression levels as compared to J + EE + A rats (p = 0.018). In the dCA1, α1 expression levels in J + A rats were significantly increased only compared to controls (p = 0.013).

In the ventral hippocampus (Fig. 4B), two-way ANOVA revealed a significant effect for both J + A and EE exposure and their interaction on GABA_A_ α1 expression levels in the DG (J + A: F(3,67) = 6.42, p = 0.013; EE: F(3,67) = 7.201, p = 0.002; Interaction: F(3,67) = 7.697, p = 0.000) and the CA1 (J + A: F(3,67) = 13.267, p = 0.001; EE: F(3,67) = 8.699, p = 0.001; Interaction: F(3,67) = 6.855, p = 0.000). Further post hoc comparisons revealed increased vDG α1 expression levels in both J + A and J + A + EE rats compared to control (p = 0.007, p = 0.000, respectively) and J + EE + A rats (p = 0.017, p = 0.000, respectively). In the vCA1, increased α1 expression levels were found in J + A rats compared to control (p = 0.001), J + EE + A (p = 0.000), and to J + A + EE (p = 0.032). Additionally, a significant increase in α1 expression levels was found in J + A + EE rats compared to J + EE + A rats (p = 0.043).

### Assessing expression levels of GABA_A_ receptor α1 subunit in dorsal and ventral hippocampal subregions following the behavioral profiling characterization

**Figure 5:**
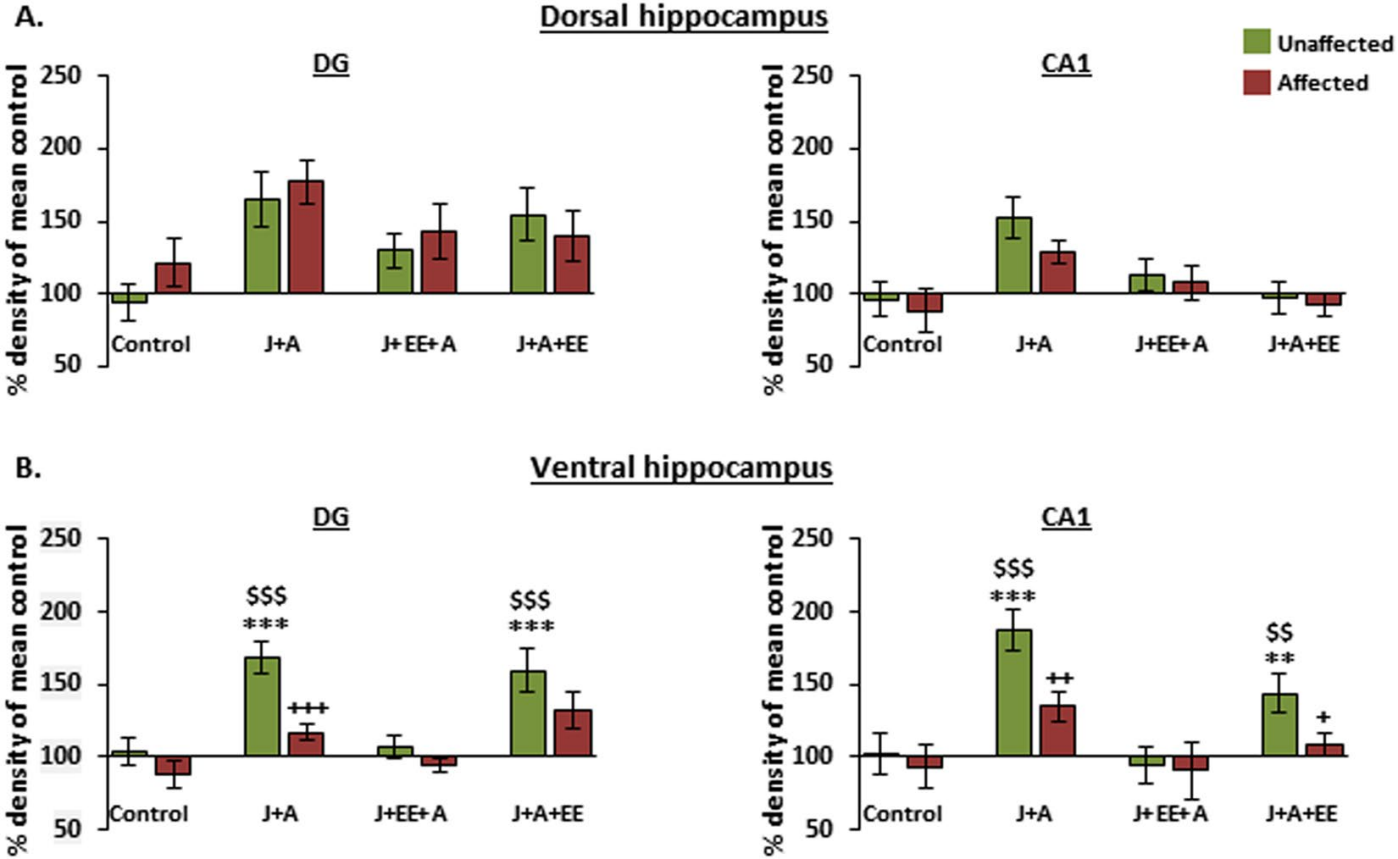
Increased expression levels of GABA_A_ receptor α1 subunit in the ventral hippocampal subregions of stress resilient animals. **(A**) Dorsal hippocampus. No significant interactions of stress-exposure group x behavioral profile were observed in either the dDG or dCA1. **(B**) Ventral hippocampus. For GABA_A_ receptor α1 in the vDG, a significant interaction of stress-exposure group x behavioral profile was observed. An increased expression of GABA_A_ receptor α1 was observed in unaffected rats of both J + A and J + A + EE groups, compared to unaffected rats from the control and J + EE + A groups. Direct comparison also revealed an increase in GABA_A_ receptor α1 levels in unaffected rats compared to affected rats within the J + A group. A similar expression pattern was observed for GABA_A_ receptor α1 in the vCA1 of unaffected rats in both J + A and J + A + EE groups, compared to unaffected rats from the control and J + EE + A groups. Here, direct comparison revealed an increase in GABA_A_ receptor α1 levels in unaffected rats compared to affected rats in both J + A and the J + A + EE groups. All values are % density of mean control (mean ± SEM). Significantly different from unaffected control: **p < 0.01, ***p < 0.001. Significant difference from unaffected J + EE + A: ^$$^p < 0.01, ^$$$^p < 0.001. Significant difference unaffected vs. affected rats: ^+^p < 0.05, ^++^p < 0.01, ^+++^p < 0.001.

Analysis of the effects of EE intervention on α1 expression levels using group averages might mask expression alterations associated with the different behavioral profiles of individuals, as previously reported^16^. We thus re-analyzed the levels of expression according to the categorization of affected vs. unaffected individuals. First, Multivariate analysis for GABA_A_ receptor α1 expression levels in each subregion was applied, assessing the interactions between exposure and behavioral profiles (unaffected vs. affected animals). In the dorsal hippocampus, two-way ANOVA did not reveal any significant interactions in either the dDG or the dCA1 (Fig. 5A: F(3,60) = 0.51, n.s. and F(3,60) = 1.07, n.s., respectively). By contrast, in the ventral hippocampus, two-way ANOVA revealed significant interactions in both the vDG and vCA1 (Fig. 5B: F(3,60) = 2.91, p = 0.041 and F(3,60) = 3.33 p = 0.025, respectively).

Second, comparisons using one-way ANOVA revealed a significant effect for Group among unaffected rats in both the vDG and vCA1 (ANOVA between groups of unaffected populations F(3,40) = 15.43, p = 0.000 and F(3,39) = 11.64, p = 0.000, respectively). In the vDG, further post hoc comparisons revealed a specific increase in GABA_A_ receptor α1 expression in unaffected rats from both J + A and J + A + EE groups compared to unaffected rats from the control (p = 0.000. p = 0.000, respectively) and J + EE + A groups (p = 0.001, p = 0.000, respectively). In the vCA1, further post hoc comparisons revealed a specific increase in GABA_A_ receptor α1 expression levels in unaffected rats from both J + A and J + A + EE groups compared to unaffected rats from the control group (p = 0.000. p = 0.007, respectively) and from J + EE + A rats (p = 0.000, p = 0.002, respectively). This was further confirmed by a direct comparison of vDG and vCA1 α1 levels in unaffected vs. affected J + A rats (T(14) = 4.483, p = 0.000 and T(13) = 6.821 p = 0.012, respectively) and vCA1 α1 levels in unaffected vs. affected J + A + EE rats (T(15) = 4.94, p = 0.026). No expression regulation was observed in affected animals (ANOVA between groups of affected populations F(3,26) = 2.14, n.s. and F(3,26) = 2.69, n.s., respectively).

## Discussion

**Figure 6:**
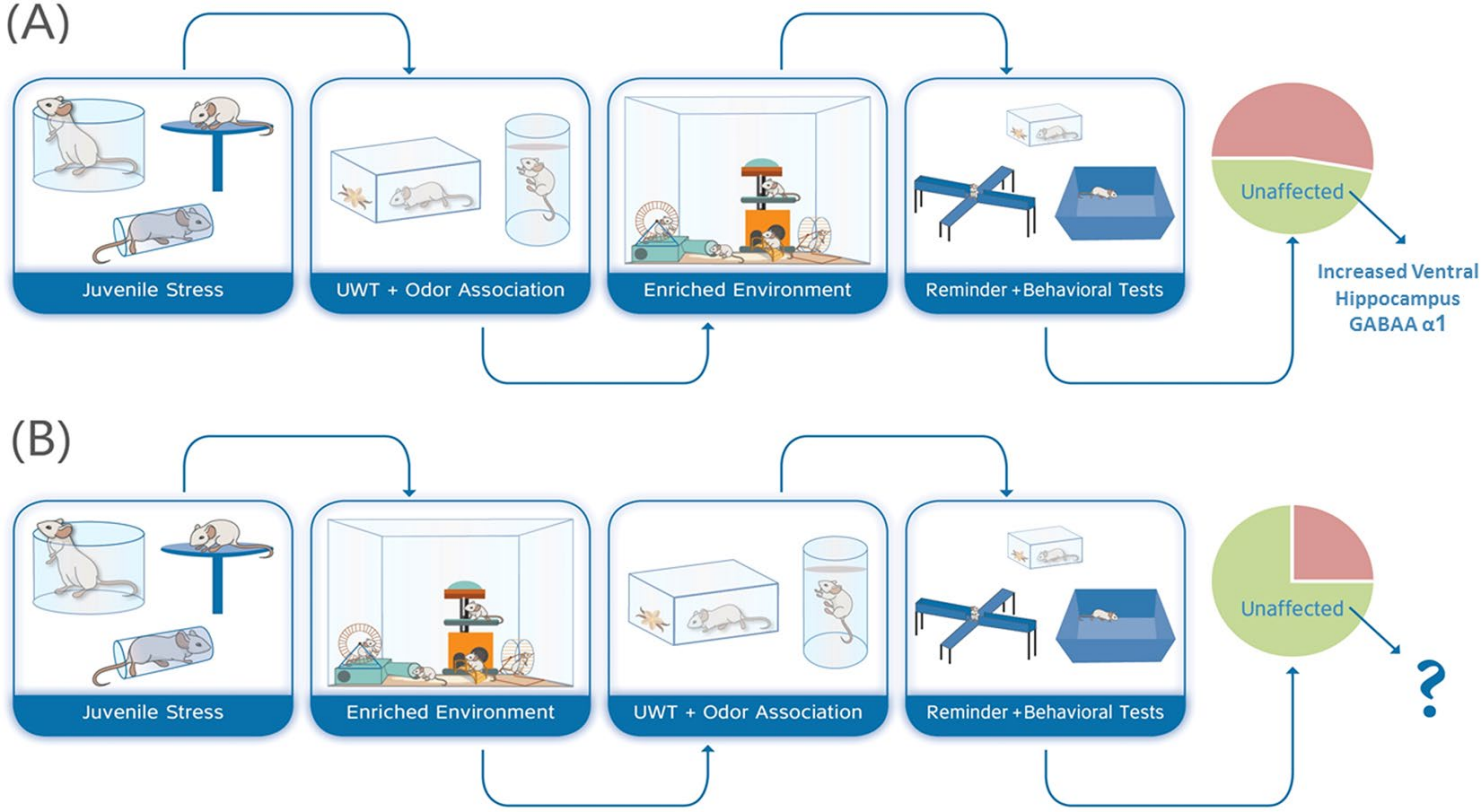
A graphic summary: EE during juvenility reduces the impact of juvenile stress, an effect that is not mediated by increased expression of GABA_A_ receptor α1. **(A)** EE for one month following exposure to J + A stress is not effective in reducing the adverse impact of the stress exposure. The proportion of affected animals is similar to the proportion of affected animals following J + A stress without exposure to EE. A significant increase in the expression of α1 in the ventral hippocampus was found in animals that were exposed to the J + A protocol and did not develop PTSD-like symptoms, as previously reported for unaffected animals without exposure to EE^18^. This result supports the notion that expression of α1 in the ventral hippocampus is associated with increased stress resilience. **(B)** EE for one month following exposure to juvenile stress was effective in reducing the negative impact of juvenile stress on coping with trauma in adulthood. However, resilience following the exposure to EE was not associated with increased expression of α1 in the ventral hippocampus, suggesting that the neural mechanisms involved in EE-associated resilience are different from those associated with naturally-occurring stress resilience.

Our results corroborate the findings that increased expression levels of the GABA_A_ receptor α1 subunit in the ventral hippocampus is associated with stress resilience^18^, using a different experimental context. Furthermore, our findings demonstrate the potential importance of early interventions, from pre-puberty and into adulthood, in building up stress resilience and in reducing the negative impact of juvenile stress on coping with trauma in adulthood. Juvenility is a stress-sensitive period during which exposure to aversive experiences increases the probability of developing psychopathologies in the face of trauma later in life^6–10,17,18^. However, juvenility may also be an effective period for initiating intervention. We have already demonstrated that selective serotonin reuptake inhibitors (SSRIs)^11^ or EE^12^ interventions can reduce the impact of juvenile aversive experiences into adulthood. Here we further demonstrate that EE later in life, from immediately following the exposure to trauma in adulthood, is no longer effective (Fig. 6).

The current results further demonstrate that EE-associated increased resilience is mediated by a mechanism that is not dependent on increased α1 expression in the ventral hippocampus. Resilience-associated increased α1 expression in the ventral hippocampus was found in unaffected individuals of the J + A and J + A + EE groups, but not in unaffected individuals of the J + EE + A group (Fig. 6).

What may mediate the effects of EE? EE has been associated with enhanced neurogenesis and increased expression of brain derived neurotrophic factor (BDNF), and those are often suggested to mediate the beneficial effects of EE on learning and memory and on stress resilience^22,23^. Exposure to stress and trauma^24^, including exposure to pre-pubertal stress^25^, is suggested to result in reduced expression of BDNF and impaired neurogenesis in the hippocampus, and EE is assumed to reverse these effects by enhancing hippocampal neurogenesis and BDNF expression^22,26–32^. In addition, EE was found to prevent the reduction in hippocampal volume in an animal model of PTSD^33^. Reduced hippocampal volume has been suggested in human PTSD together with the possibility that it may result from the impaired neurogenesis associated with exposure to stress and trauma^24^. Similarly, chronic stress exposure, a well-established model of mood disorders, results in mood-related symptoms reminiscent of depressive symptoms in mood disorders^34^. In addition, antidepressants such as SSRIs were found to have effects similar to EE, including increased hippocampal neurogenesis and BDNF expression^34^. Together, these findings have led to the conception that upregulation of BDNF and subsequent induction of neurogenesis mediate the effects induced by both SSRIs and EE in stress-related and mood disorders^34,35^, and that hippocampal neurogenesis plays a major role in stress resilience^23^.

There are, however, opposing observations, which indicate that hippocampal neurogenesis is not required for at least some behavioral effects of enriched environment^36,37^ or for some effects of anti-depressants^38,39^. Furthermore, resilience is found also without exposure to EE or to SSRIs^18^. These findings point to the involvement of additional factors.

Such proposed additional factors are alterations in the activity of GABAergic interneurons, which are important modulators of neural activity and plasticity. Alterations in GABAergic neurotransmission have been implicated in stress, trauma, and PTSD^14–17^. PTSD has been found to be associated with reduced GABA_A_ benzodiazepine receptor binding^40^, and although the use of benzodiazepines as medication in PTSD is under debate, these drugs are nevertheless widely prescribed to PTSD patients^41^. The effects of EE on neurogenesis have been found to involve effects on GABAergic interneurons^42,43^. Furthermore, BDNF, a key mediator of protective EE effects after stress^22,23^, has been found to be involved in modulating GABAergic neurotransmission in relation to both stress vulnerability and stress resilience^44,45^. Stress-induced alterations in neurosteroid biosynthesis and changes in GABA_A_ receptor subunit expression have already been suggested as potential biomarkers and targets for the development of new PTSD treatments^46^. Interestingly, pre-pubertal stress has been found to alter the expression of both neurosteroids^47^ and GABA_A_ receptor α subunits expression^18,48,49^. The current results, indicating that alteration of the expression of the GABA_A_ receptor α1 subunit specifically in resilient individuals, further emphasize the contribution of altered GABAergic neurotransmission in trauma-related vulnerability and resilience^44^.

The resilience-associated alteration in α1 expression in our study was relatively selective to the ventral hippocampus. This result supports our previous findings describing a selective upregulation of the GABA_A_ receptor α1 subunit in the ventral hippocampus of resilient animals^18^. The hippocampus is suggested to exert differential contributions to cognition and emotionality along its septo-temporal axis in humans, reflecting the hippocampal dorsal-ventral axis in rodents. For example, the dorsal hippocampus has been implicated in cognitive functions such as spatial learning and memory, while the ventral hippocampus has been associated with emotional responses and in particular with responses to stress^50^. Indeed, stress-induced alterations are frequently more pronounced in the ventral hippocampus^51^.

The ventral hippocampus modulates the hypothalamo-pituitary-adrenocortical axis response^52,53^, presumably via the ventral subiculum^3,54^. Exposure to chronic stress affects neurogenesis predominantly in the ventral hippocampus^55^. It has been suggested that the effects of antidepressants on BDNF, neurogenesis, and behavior are mediated mainly by their effects on the ventral hippocampus^34^. More specifically, it is suggested that preferential regulation of neurogenesis in the ventral hippocampus contributes to stress resilience and antidepressant action^23,56,57^. Thus, a selective upregulation of the GABA_A_ receptor α1 subunit in stress resilient animals may comprise an additional mechanism of emotion-related changes of the ventral hippocampus. These resilience-related mechanisms can presumably be achieved more effectively during juvenility, adolescence, and into young adulthood, as previously suggested^11,12^.

Examining individual differences with regards to stress vulnerability and resilience appears to be critical for our understanding of the neural basis of stress-related psychopathology and stress resilience. The current results, together with previous findings^17,18,23,58^, emphasize that exposure to trauma, which may lead to the development of stress-related psychopathologies, such as PTSD, involves the activation of numerous neuronal responses and alterations. Some of these alterations presumably underlie the development of pathology. However, some of these changes rather reflect attempts of the system to regain control and support stress-resilience^17^. Dissociating between vulnerability/pathology-associated alterations and those associated with stress resilience is clearly imperative for successful translation of preclinical findings into effective treatment. Introducing behavioral profiling analysis and distinguishing between exposed-affected and exposed-unaffected individuals is an important tool in that respect, which here led to the more accurate association of the elevated expression of the GABA_A_ receptor subunit α1 with stress resilience. Assessing the impact of EE manipulation on the proportion of affected versus unaffected individuals rather than on the averaged group results helped demonstrate the relative effectiveness of pre-puberty EE intervention compared to the impact of a similar manipulation following exposure to trauma in adulthood. Exposure to aversive experiences in childhood is a known risk factor for developing psychopathologies later in life^1–3^. The current findings emphasize the importance of considering early interventions, during pre-puberty, in order to reduce the likelihood of developing psychopathologies later in life.
